# Behavioral repertoire of high‐shore littorinid snails reveals novel adaptations to an extreme environment

**DOI:** 10.1002/ece3.7578

**Published:** 2021-05-02

**Authors:** Terence P. T. Ng, Sarah L. Y. Lau, Mark S. Davies, Richard Stafford, Laurent Seuront, Neil Hutchinson, Tommy T. Y. Hui, Gray A. Williams

**Affiliations:** ^1^ The Swire Institute of Marine Science and School of Biological Sciences The University of Hong Kong Hong Kong SAR China; ^2^ Applied Sciences University of Sunderland Sunderland UK; ^3^ Department of Life and Environmental Sciences Bournemouth University Poole UK; ^4^ Laboratoire d’Océanologie et de Géosciences (LOG) UMR 8187 CNRS Univ. Lille Univ. Littoral Côte d’Opale Wimereux France; ^5^ Department of Marine Resource and Energy Tokyo University of Marine Science and Technology Minato Japan; ^6^ Department of Zoology and Entomology Rhodes University Grahamstown South Africa; ^7^ Tropical Futures Institute/TropWATER—Centre for Tropical Water and Aquatic Ecosystem Research James Cook University Singapore

**Keywords:** activity budget, *Echinolittorina malaccana*, *Echinolittorina radiata*, intertidal, snail, thermal stress, thermoregulation, trail following

## Abstract

Species that inhabit high‐shore environments on rocky shores survive prolonged periods of emersion and thermal stress. Using two Hong Kong high‐shore littorinids (*Echinolittorina malaccana* and *E*. *radiata*) as models, we examined their behavioral repertoire to survive these variable and extreme conditions. Environmental temperatures ranged from 4°C in the cool season to 55.5°C in the hot season, with strong seasonal and daily fluctuations. In the hot season, both species allocated >35% of their activity budgets to stress‐mitigating thermoregulatory behaviors (e.g. standing, towering) and relatively small proportions to foraging (<20%) and reproduction (<10%). In the assumedly benign cool season, greater proportions (>70%) of activity budgets were allocated to stress mitigation behaviors (crevice occupation, aggregation formation). Both species exhibited multifunctional behaviors that optimized time use during their tidally‐constrained activity window in the hot season. Females mated while foraging when awash by the rising tide, and some males crawled on top of females prior to ceasing movement to form 'towers', which have both thermoregulatory benefits and reduce searching time for mates during subsequent activity. The function of such behaviors varies in a state‐dependent manner, for example, the function of trail following changes over an activity cycle from mate searching on rising tides, to stress mitigation on falling tides (aiding aggregation formation), and to both functions through tower formation just before movement stops. Many of these behavioral responses are, therefore, multifunctional and can vary according to local conditions, allowing snails in this family to successfully colonize the extreme high‐shore environment.

## Graphical abstract

High shore littorinids experience strong variations in temperature on seasonal and daily bases. Within these regimes, they show multifunctional behaviours that have thermoregulatory benefits. The function of such behaviours varies in a state‐dependent manner.

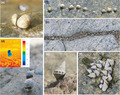

## 1 INTRODUCTION

Physiological tolerance plays a critical role in the distribution limits of species in the intertidal zone. Porcelain crabs living high on the shore, for example, have greater upper thermal tolerances than lower shore species (Stickle et al., 2017; Stillman, 2002; Stillman & Somero, 2000). Marine species living at the upper levels of the intertidal zone ('high‐shore species' hereafter) often persist in what are essentially terrestrial conditions for days or even weeks (Marshall & McQuaid, 2011; Marshall, McQuaid, et al., 2010; McMahon, 2001; McQuaid, 1996; Ng et al., 2017). On tropical rocky shores these high‐shore species often experience environmental (rock) temperatures >55°C in the hot season (Marshall et al., 2013; Williams & Morritt, 1995) and are remarkably tolerant to heat stress (e.g. Lee & Lim, 2009; Liao et al., 2017; Marshall et al., 2011; McMahon, 1990). High‐shore species are also assumed to live close to their upper thermal limits (Stillman, 2002; Stillman & Somero, 2000) and seem to have evolved high heat tolerance at the expense of their physiological and biochemical acclimation capacities, making them vulnerable to increasing temperatures (Somero, 2010; Stillman, 2003).

A suite of behavioral adaptations, however, enable mobile high‐shore species to maintain a wider thermal safety margin, maintaining their body temperatures below lethal thermal limits, than species found lower on the shore (Ng et al., 2017). High‐shore littorinids, for instance, have exceptional thermal tolerances (Liao et al., 2017; McMahon, 2001; Ng et al., 2017); can depress metabolism during severe heat stress (Marshall & McQuaid, 2011; Marshall, McQuaid, et al., 2010); and also adopt various stress‐mitigating, thermoregulatory behaviors that enhance their ability to withstand extreme thermal environments (Garrity, 1984; Hayford et al., 2015; Marshall & Chua, 2012; Seuront & Ng, 2016). Marshall et al. (2015), in particular, noted that these snails have a potentially narrow thermal safety margin but behavioral thermoregulation can provide considerable buffering capacity.

Stress‐mitigating behaviors play a key role in the survival of mobile intertidal species when thermal and desiccation stresses become severe during emersion periods (Garrity, 1984; Harper & Williams, 2001; Hayford et al., 2015; Reid & Harley, 2021; Williams & Morritt, 1995). Most species are adapted to time their periods of activity and inactivity to minimize exposure to thermal stress. In the tropics this usually involves moving to feed and reproduce while awash by the tide and then adopting various stress‐mitigating behaviors such as sheltering in cool refuges while emersed (Garrity, 1984; Hutchinson & Williams, 2001; Williams & Little, 2007; Williams & Morritt, 1995). The time individuals allocate to stress mitigation is, therefore, strongly influenced by environmental variation which can impose a limit on the time allocated to other key activities (e.g. foraging and reproduction), with subsequent consequences for fitness (Dunham et al., 1989; Frid & Dill, 2002). The ability to maximize fitness through strategic partitioning of different activities can be crucial in determining species’ life‐history traits and hence success in a given environment (Dunham et al., 1989; Frid & Dill, 2002; Gunderson & Leal, 2015).

Snails in the family Littorinidae, being abundant and important herbivores in high‐shore environments worldwide (reviewed by McMahon, 1990, 2001; McQuaid, 1996; Reid, 1989), are excellent models to investigate the strategic partitioning of behaviors that allow successful utilization of these extreme habitats. These snails exhibit various behaviors common to many mobile intertidal ectotherms such as trail following, refuge selection, aggregation, and as they undergo internal fertilization, mating and fighting for mates (see Cartwright & Williams, 2012; Ng et al., 2013, 2016; Stafford et al., 2007). They also perform shell‐posturing behaviors, for example standing and towering (Marshall & Chua, 2012; Marshall, Mustafa, et al., 2010; Seuront & Ng, 2016). Some of these behaviors are known to have multiple functions; trail following, for example, is associated with foraging, mating, energy saving, and aggregation formation (reviewed by Ng et al., 2013).

Most previous studies (e.g. Cartwright & Williams, 2012; Ng et al., 2013, 2016; Seuront & Ng, 2016; Stafford et al., 2007) have, however, only investigated the importance of a single function at a given time and have not considered how these snails can vary their behavioral repertoires to optimize the partitions between energy gain, reproduction, and amelioration of thermal stress in the extreme conditions of the high shore. To address this, we investigated variation in the behavioral repertoire, in terms of activity budget and utilization of multifunctional behaviors, of two high‐shore species, *Echinolittorina malaccana* and *E*. *radiata*, in Hong Kong. Hong Kong has a seasonal, monsoonal climate with a benign cool (and dry) winter season and a stressful hot (and wet) summer (Chan et al., 2006; Kaehler & Williams, 1996; Nagarkar & Williams, 1999). On account of this strong seasonal shift in environmental conditions, we hypothesized that the activity budget of snails would vary between seasons; specifically that snails would allocate proportionately more time to foraging and reproduction in thermally benign conditions (the cool season), and to stress‐mitigating behaviors (e.g. standing; towering; seeking refuge in crevices; aggregating) in thermally stressful conditions (the hot season). Due to the constrained duration of potential activity periods, we also assessed how different behaviors are utilized dependent on environmental conditions and considered how they may potentially contribute to survival in extreme and strongly fluctuating thermal environments.

## 2 METHODS

### 2.1 Study site

The behavioral patterns of the littorind snails *Echinolittorina malaccana* and *E*. *radiata* were investigated between 2014 and 2016 on semi‐exposed rocky shores in the Cape d’Aguilar Marine Reserve, Hong Kong (22°12′27″N, 114°15′36″E), at four (data pooled across sites to ensure adequate sample sizes) sites (each 6–20 m long; separated by barriers such as sand or boulders, or located more than 10 m apart) where these snails co‐occur and are abundant in the high shore (Mak, 1996; Mak & Williams, 1999). In the hot season (June to October, see Kaehler & Williams, 1996), the vertical ranges of the species largely overlap, forming a high‐density band with *E*. *malaccana* slightly higher on the shore than *E*. *radiata* and exhibiting a correspondingly higher thermal tolerance (LT_50_ in air during summer = 56.5 and 55.5°C, respectively, Li, 2012). In contrast, in the cool season (December to April), when temperatures may lead to reduced speed but are not cold enough to limit activity of the snails (SLY Lau, unpublished data), there is less overlap, and although both species migrate up‐shore, *E*. *malaccana* is found consistently higher on the shore than *E*. *radiata* (Mak, 1996).

### 2.2 Thermal environment

During Hong Kong's hot season, spring low tides occur in the afternoon, when solar irradiation is high; whereas in the cool season they occur in the early morning (Kaehler & Williams, 1996). To measure the thermal environment, temperature loggers (Thermochron iButton DS 1922L, Maxim Integrated, USA) sampling at hourly intervals were deployed in the resting zone (areas that the snails occupied during emersion) of the two species at two sites with abundances >400 individuals m^‐2^. At each site three waterproofed (embedded in Scotchcast model 2131 resin (3M, USA) within the cap of a Falcon tube, following Marshall et al., 2013) loggers were fixed to the rock using epoxy resin at 2.2–2.3 m above Chart Datum (CD) in the hot season (June to August 2015) and at 2.6–2.7 m above CD in the cool season (January to March 2016). As these loggers were separated from the rock by a thin layer of epoxy, they provided an index of the rock temperature experienced on the shore.

### 2.3 Activity budget

**FIGURE 1:**
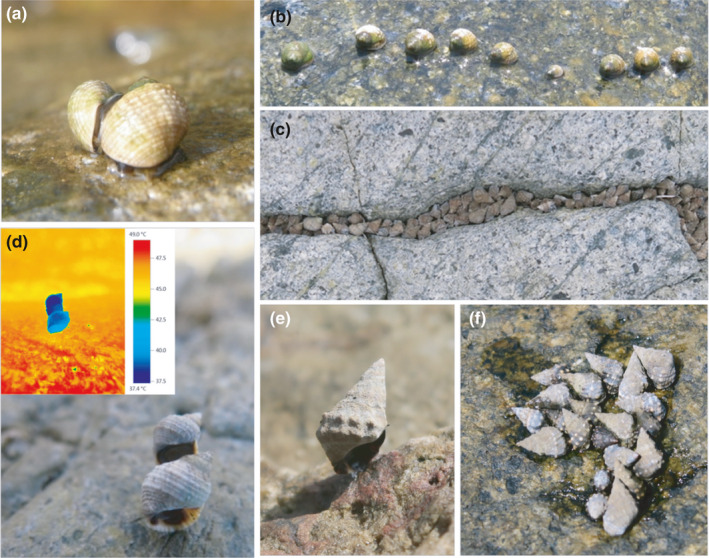
Behavioral repertoire of *Echinolittorina malaccana* (EM) and *E*. *radiata* (ER): a mating pair of ER (a); trail‐following behavior in ER (b); both species sheltering in a crevice (c); towering behavior in ER (inset: thermal image showing the cooling effect of the tower) (d); standing behavior in EM (e); and an EM aggregation that retains water (f)

Hourly surveys lasting 24 hr were conducted during spring tides to investigate the proportion of snails exhibiting various behaviors (Table 1; Figure 1) associated with different fitness‐associated activities (i.e. foraging, reproduction, and stress mitigation) on days with typical thermal patterns in the hot (August 2015, hot and without rain with average daily maximum rock temperatures >40°C) and cool (February 2016, cool and without rain with average daily maximum rock temperatures <30°C) seasons at one site. There was no within‐season replication of these surveys, which limits interpretation of variation within seasons owing to small‐scale weather patterns, but does allow assessment of larger‐scale differences between seasons. Each hour ten 25 × 25 cm quadrats were haphazardly placed in the zone where the two species occurred and the number of individuals exhibiting different behaviors recorded (Table 1; Figure 1). Behaviors scored and their assumed fitness‐associated activities were based on previous studies of species in the same family (Littorinidae, Table 1). Individuals performing multiple behaviors simultaneously related to the same fitness‐associated activity (e.g. aggregation and sheltering in crevices, Figure 1c; or standing and towering, Figure 1d) were only scored once to avoid bias toward any particular activity. Since it was not possible to observe when individuals were grazing on the shore biofilm, we ascribed any movement to foraging (after Hartnoll & Wright, 1977; Little et al., 1988; since in general snails rasp the rock surface when they move). Due to both the difficulty in simultaneously scoring multiple behaviors when snails were awash in the hot season and in identifying the multifunctional nature of trail‐following behavior (Ng et al., 2013), we did not record trail‐following behavior in awash quadrats, but conducted a separate study on this behavior (see below). In the hot season, the height of the high‐density band of snails moving while awash was measured hourly, recording the height above CD at the middle of the band using a cross‐staff (see Little et al., 2009).

**TABLE 1:** Descriptions of various fitness‐associated activities and behaviors of *Echinolittorina malaccana* and *E*. *radiata*

Fitness‐associated activities	Behaviors	Description
Reproduction	Mating	An individual (male) mounts the other individual's (female's) shell in an anticlockwise manner, eventually reaching the right‐hand side where the male copulates with the female by inserting its penis into the female's mantle cavity (Figure 1a)^a^
	Fighting	When two individuals (males) simultaneously mount the same individual (female) and the males push against each other, or when one male (the defender) is copulating with a female and another (the challenger) encounters the pair and attempts to push away the defender^b^
Foraging	Moving	In <5 s observation, snails crawled
	Turning	In <5 s observation, snails rotated in‐place without any distance traveled
Stress mitigation	In crevices	Snails residing in pits and holes in rock (Figure 1c)
	Towering	Snails climbing and settling on others to form a stack (Figure 1d)^c^
	Standing	A snail whose shell is attached to the substratum via a mucus holdfast, so that the body is lifted from the substratum with the aperture positioned perpendicular to the substratum (Figure 1e)^d^
	Aggregating	Three or more snails in direct contact with each other (Figure 1f)^e^
Inactive	Stationary	In >5 s observation, snails showing no motion or other activity, and not exhibiting any stress mitigation behaviors

^a^ Gibson (1965), Saur (1990), Ng and Williams (2014).

^b^ Gibson (1965), Ng et al. (2016).

^c^ Marshall, Mustafa, et al. (2010)), Seuront and Ng (2016).

^d^ Lim (2008), Marshall and Chua (2012), Seuront and Ng (2016).

^e^ Chapman (1995), Stafford and Davies (2004).

### 2.4 Multifunctional behavior: trail following for mating and/or for stress mitigation

Trail‐following behavior occurred when snails were awash by the tide and involved an individual (a tracker) moving along the trail of another (a marker) individual for >5 s (Davies & Beckwith, 1999). In the hot season this behavior in most cases involved two individuals during rising tides and multiple individuals during falling tides. Snails involved in trail following were sampled at all four sites to test whether the function of the behavior changed from finding a mate on the rising tide (i.e. males following females; see Ng et al., 2011, 2013, for more details), to finding and forming an aggregation on the falling tide (i.e. trail following regardless of sex and species; Stafford et al., 2007). At each site in the hot season of 2014 (July and August), a minimum of 30 trail‐following pairs of each species along different trails were haphazardly collected on both the rising (tidal height: ~1.7 to 2.0 m +CD) and the falling tide (~2.0 to 1.7 m +CD). For *E*. *malaccana*, the pairs were collected during one rising tide and one falling tide within the same day, whereas those for *E*. *radiata* were collected during one rising tide and one falling tide within 2 days. In cases where animals were trail following in a chain of three or more, only the two leading animals were sampled. Snails were transferred to the laboratory and sexed, based on the presence of a penis. We considered it extremely unlikely that we had mistaken female snails for immature males because: juveniles recruit in the cool season and usually reach sexual maturity (with shell length ~>3 mm) before the hot season (Mak, 1996); and mating males of *E*. *malaccana* have been recorded at 3.2 mm and of *E*. *radiata* at 2.5 mm (Ng et al., 2019), and all the females we identified as such in this study were >4 mm, except one *E*. *malaccana* at 2.9 mm. Sampling was not conducted at high tide, because animals were either stationary or moving slowly and trail following was not apparent. Repeated sampling on each shore on both rising and falling tides might have affected snail behavior, but as densities were so high we were able to sample snails without affecting other individuals and so considered this potential effect to be negligible.

To test whether trail‐following pairs were dominated by a particular marker–tracker combination (male followed by a male, female followed by a male, etc.) on rising and falling tides, expected numbers of these different combinations were calculated assuming random pairing and tested against the observed numbers using a chi‐squared test. Specifically, if there are *m* males and *n* females and, based on a random pairing scenario where each individual has a 50% chance of being either a marker or follower, the expected numbers of FM, MM, MF, and FF (where XY denotes Y follows X; e.g. MF = male followed by a female) are n*0.5*(*m*/(*n* + *m* − 1)), m*0.5*((*m* − 1)/(*n* + *m* − 1)), m*0.5*(*n*/(*n* + *m* − 1)), and n*0.5*((*n* − 1)/(*n* + *m* − 1)), respectively. Data from all four sites were pooled for each species on rising (*E*. *malaccana*: *N* = 126 pairs. *E*. *radiata*: *N* = 120 pairs) and falling tides (*E*. *malaccana*: *N* = 120 pairs. *E*. *radiata*: *N* = 120 pairs).

### 2.5 Multifunctional behavior: towering for mating and/or for stress mitigation

Sampling was conducted during the hot season of 2015 (June) to investigate whether towering behavior functioned as a strategy to facilitate mating in addition to aiding thermoregulation (Marshall, Mustafa, et al., 2010; Seuront & Ng, 2016). Over 5 days when towering occurred, >40 conspecific towers of each species were collected from the four sites during daytime both during emersion before activity began on the rising tide (*E*. *malaccana*, *N* = 41; *E*. *radiata*, *N* = 40) and when activity ceased on emersion on the falling tide (*E*. *malaccana*, *N* = 64; *E*. *radiata*, *N* = 43). The position (top or bottom) of individuals in each tower was noted prior to collection, and each individual was sexed on return to the laboratory. Data were pooled across sites and days. Only conspecific towers that consisted of two individuals were sampled because these towers were by far the most abundant on the shore (Seuront & Ng, 2016; Figure 1). For sampling conducted before the rising tide, individuals were collected after they were wetted by the tide. As towers were wetted, the topmost individual dismounted the other and it was noted whether this individual then initiated mating (defined as when it had maintained the mating position for >20 s) or moved away from the other animal.

To test whether the towers were dominated by a particular sex combination (male on top of male, male on top of female, etc.), chi‐squared tests were performed to test the observed frequencies of the four sex combinations against the expected frequencies, which were calculated assuming random pairing (expected numbers calculated as described above).

## 3 RESULTS

### 3.1 Thermal environment

**FIGURE 2:**
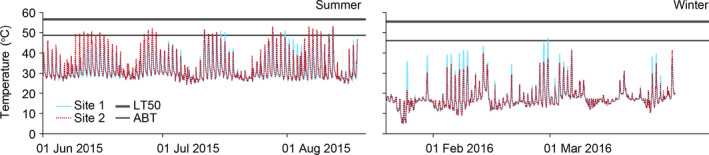
Examples of seasonal fluctuations (79 days in the hot, summer season and 73 days in the cool, winter season) in temperature (mean of three iButton data loggers at two sites, S1 and S2) in Cape d' Aguilar Marine Reserve, Hong Kong. The Arrhenius Breakpoint Temperature for heart rate (thin line) and LT_50_ (bold line) values (averaged of the two study species, after Li, 2012) are indicated by the horizontal lines

Ambient temperatures ranged from 24.1 to 55.5°C in the hot season with ~25% and 42% of day temperatures exceeding 45°C in Sites 1 and 2, respectively, and from 4.0 to 49.1°C in the cool season with ~8% and 3% of days temperatures exceeding 40°C in Sites 1 and 2 (Figure 2).

### 3.2 Activity budget

**FIGURE 3:**
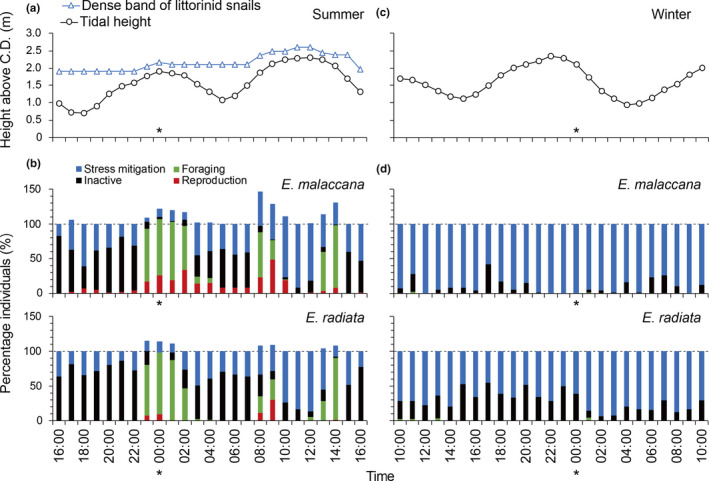
Tidal height (a and c) and hot season (summer) vertical movement (a) of snails across a 24‐hr tidal cycle with the percentage of each species performing different behaviors (see Table 1) each hour (b and d, note time axes are different and midnight is marked by asterisks) in the hot (summer) and cool (winter) seasons. Note the total number of behaviors can exceed the total number of snails if snails perform multiple behaviors (hence maximum % individuals can exceed 100%)

Both species spent most of their time 'inactive' (*sensu* Table 1) or exhibiting stress mitigation behaviors, especially in the cool, assumedly benign, season, where these behaviors accounted for >90% of activity budgets (Figure 3). In the cool season, both species stayed close to the corresponding daily high water level, consistently aggregating in crevices, with *E*. *radiata* spending more time inactive on bare rock than *E*. *malaccana* (Figure 3). No shell‐posturing behaviors (standing or towering) or reproductive activities and almost no foraging (<1% activity budget) were recorded for either species (Figure 3).

In the hot season, individuals of both species moved up and down the shore with the tide. Inactivity and stress mitigation behaviors (standing, towering, aggregation, and crevice occupation) occurred mainly during the falling tide and emersion periods (Figure 3). When the tide was rising in the hot season, individuals engaged in foraging and reproductive activities (i.e. mating and fighting, Figure 3). Fewer *E*. *radiata* showed reproductive activities than *E*. *malaccana* and *E*. *radiata* also stopped these activities earlier than *E*. *malaccana* during the awash phase, even though both species allocated a similar proportion of their activity budget to foraging (17%–19%; Figure 3).

Individuals often performed more than one behavior associated with more than one fitness‐associated activity (resulting in sums of >100% activity for individuals at certain times, especially in the hot season; Figure 3). Such cases included (a) females mating (reproduction) and moving/turning (foraging) simultaneously (these females moved noticeably more slowly than unladen females), (b) individuals mating (reproduction) while forming aggregations (stress mitigation), and (c) individuals aggregating (stress mitigation) while turning (foraging).

Overall, our hypothesis that snails would allocate proportionately more time to foraging and reproduction in thermally benign conditions (the cool season), and to stress‐mitigating behaviors in thermally stressful conditions (the hot season) was not supported. While snail behavior was consistent with individuals remaining largely inactive in crevices and aggregations in the cool season, in the hot season the picture was more complex, whereby some behaviors, specifically trail following and towering, appeared to serve more than one function.

### 3.3 Multifunctional behaviors

**FIGURE 4:**
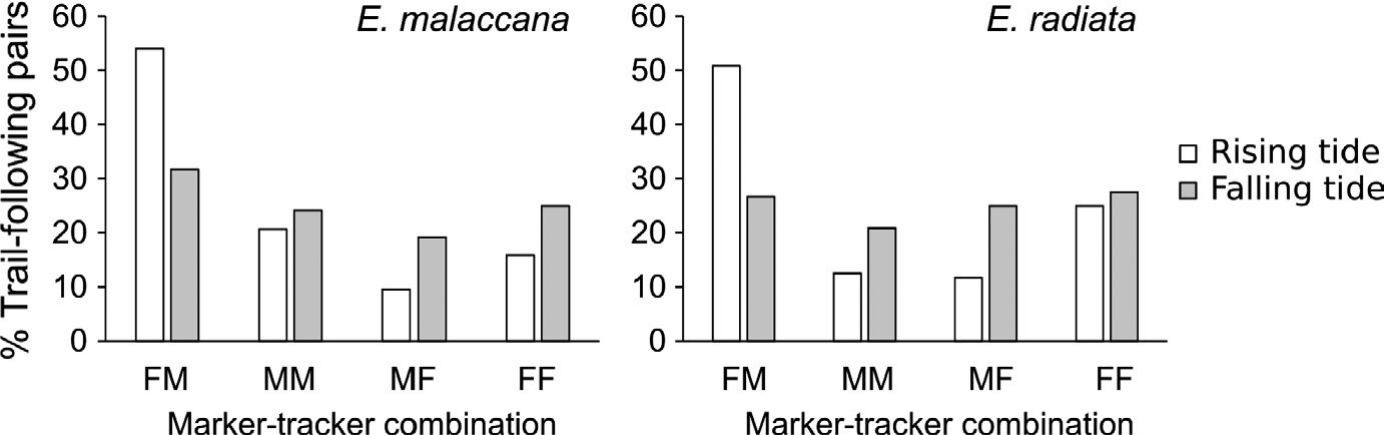
Percentage trail‐following pairs of the four marker–tracker combinations observed on rising and falling tides in *E*. *malaccana* and *E*. *radiata* pooled from four sites. FM, female followed by male; MM, male followed by male; MF, male followed by female; FF, female followed by female

During the rising tide, trail‐following pairs were not randomly formed, with the most dominant marker–tracker combination being males following females (54% and 51% in *E*. *malaccana* and *E*. *radiata,* respectively; Table 2; Figure 4). During the falling tide, however, the sex of trail‐following pairs was randomly distributed (Table 2; Figure 4).

**TABLE 2:** Number of each different male–female marker–tracker combination in trail‐following pairs sampled on rising tides and falling tides for *Echinolittorina malaccana* (*N* = 126 pairs on rising tides and *N* = 120 pairs on falling tides) and *E*. *radiata* (*N* = 120 pairs on rising tides and *N* = 120 pairs on falling tides)

	Marker	Tracker	Observed	Expected
*E. malaccana*				
On rising tides	Female	Male	68	31.6
	Male	Male	26	34.4
	Female	Female	20	28.4
	Male	Female	12	31.6
			χ^2^ = 58.79, *p* < .001	
On falling tides	Female	Male	38	30.1
	Male	Male	29	29.4
	Female	Female	30	30.4
	Male	Female	23	30.1
			χ^2^ = 3.75, *n*.s.	
*E. radiata*				
On rising tides	Female	Male	61	29.7
	Male	Male	15	22.8
	Female	Female	30	37.8
	Male	Female	14	29.7
			χ^2^ = 45.72, *p* < .001	
On falling tides	Female	Male	32	30.0
	Male	Male	25	26.0
	Male	Female	30	30.0
			χ^2^ = 0.20, n.s.	

Note

Chi‐squared tests (*df* = 3 in all cases) report observed frequencies against expected frequencies where trail‐following pairs form at random.

Towers of both species collected before the rising tide and after the falling tide were also not randomly formed, consisting mainly of a male on top of a female (overall ~60%, Table 3). This phenomenon was more pronounced in *E*. *radiata* and before the rising tide (Table 3). All pairs of *E*. *malaccana*, and 55.6% of *E*. *radiata*, in conspecific towering pairs that consisted of a male on top and a female at the bottom, mated after becoming awash on the rising tide. No mating was observed when the individual on the top was a female or when the bottom individual was a male. Both trail‐following behavior and towering behavior, therefore, appear to function both in reproduction and in stress mitigation in a tidal state‐dependent manner.

**TABLE 3:** Number of towers with each sex at the different positions (top or bottom) sampled before the rising tide and after the falling tide in *Echinolittorina malaccana* (*N* = 41 before rising tide and *N* = 64 after falling tide) and *E*. *radiata* (*N* = 40 before rising tide and *N* = 43 after falling tide)

	Top	Bottom	Observed	Expected
*E. malaccana*				
Before rising tide	Male	Female	31	10.3
	Male	Male	2	8.7
	Female	Male	3	10.3
	Female	Female	5	11.7
			χ^2^ = 55.58, *p* < .001	
After falling tide	Male	Female	23	16.1
	Male	Male	17	15.9
	Female	Male	7	16.1
	Female	Female	17	15.9
			χ^2^ = 8.25, *p* < .05	
*E. radiata*				
Before rising tide	Male	Female	36	10.1
	Male	Male	2	9.9
	Female	Male	0	10.1
	Female	Female	2	9.9
			χ^2^ = 88.79, *p* < .001	
After falling tide	Male	Female	25	10.7
	Male	Male	10	13.8
	Female	Male	4	10.7
	Female	Female	4	7.8
			χ^2^ = 26.37, *p* < .001	

Note

Chi‐squared tests (*df* = 3 in all cases) report observed frequencies against expected frequencies where towers are formed by random pairing.

## 4 DISCUSSION

Although there was no assessment of within season variation in this study, there were strong seasonal and tidal variations in the behavioral budgets of the two high‐shore littorinids. There are clear seasonal differences in both phenology of the species, which are reproductively active in summer (Mak, 1996), and physiological acclimatization, with species showing slightly greater thermal tolerance (Li, 2012) in summer than winter. During the hot season, snails of both species performed 'multifunctional' behaviors, such as trail following and towering behaviors (see below), to optimize their use of time during the constrained activity window they experience living on the high shore. During the cool season however, there was little movement or foraging and snails of both species mainly aggregated in crevices.

A high proportion (*E*. *malaccana*: ~67% in summer and ~91% in winter; *E*. *radiata*: ~56% in summer and ~72% in winter) of the activity budgets of both species was allocated to stress mitigation, reproduction, and foraging, with the remainder classified as inactive (Table 1). The allocations of these behaviors are context‐dependent, varying between season and with the tidal cycle, presumably as strategies to accommodate the dynamic and challenging high‐shore environment. Given the limited time window for activity at this shore level, species should partition behaviors efficiently in time to maximize their fitness (Hughes, 1988). Nevertheless, trade‐offs may occur among decisions to perform various behaviors. Trade‐offs between stress mitigation and other fitness‐associated activities may, for example, have important sublethal consequences, as they will alter energy acquisition and expenditure (Gunderson & Leal, 2015, 2016).

One of the most parsimonious strategies in stressful environments is to utilize traits or behaviors that can serve more than one function, and therefore minimize time and/or energetic trade‐offs between functions (Hui et al., 2019; Sherbrooke et al., 2007; Song et al., 2013; Yao et al., 2010). Female littorinids in this study, for example, were able to maintain feeding while mating and so avoided a possible trade‐off in times spent between these behaviors during their constrained activity period (although there may be some cost as females that carried males appeared to move at a slower speed than unladen females). Similarly, towering behavior blurs the choice between finding a thermal refuge and finding a mate, achieving both and reducing the need for any trade‐off as on hot days the topmost snail gains a reproductive advantage and reduces its thermal stress. Indeed, since males are the active partner seeking females to mate (Figure 4; Ng et al., 2013), there may be an advantage to females and little apparent cost in allowing themselves to be at the base of towers (for example, we observed no dislodgement of the towers by waves). Towering has previously been identified as a thermoregulatory behavior in littorinid snails (Marshall, Mustafa, et al., 2010; Seuront & Ng, 2016), but also facilitates reproduction as shown in this study (see also Marshall & Ng, 2013). Males on top of the tower, for example, benefit from being cooler (by up to 10.3°C, Seuront & Ng, 2016) during emersion while reducing the time required to search for a mate when wetted by the rising tide. Clearly there is an evolutionary advantage to adopting a trait that can serve multiple functions with minimal costs (Friedman et al., 2019; Liao et al., 2019; Ng et al., 2017).

Previous studies have documented various functions of trail following in gastropods (reviewed by Ng et al., 2013). This is, however, the first study showing that gastropods adopt different functions of this behavior over a short time scale (i.e. within a tidal cycle). Males followed mainly conspecific females during rising tides, demonstrating in common with other littorinids and many other gastropods, that they recognize mucus trails laid by their prospective mates (reviewed by Ng et al., 2013). During the falling tide, however, individuals followed each other regardless of sex to aid aggregation formation and/or to save energy (Davies & Blackwell, 2007; Stafford et al., 2007). The multifunctional role of trail following may explain why different combinations of trail‐following pairs were recorded on rising and falling tides. For some snails, there appears to be a third phase of trail‐following activity in addition to 'for mating' on the rising tide and 'for stress mitigation' on the falling tide. That males preferentially occupy the top position in two‐snail, two‐sex, conspecific towers after a falling tide indicates a switch again to trail following for mating as the rock dries, particularly in *E*. *malaccana*, although the mechanisms responsible for this are unknown.

The reproductive activities of both species were also temporally partitioned: in the hot season mating occurred when snails were awash during the rising tide and stopped when the tide started to fall. *E*. *malaccana* allocated more time to reproduction (they stopped mating and moved down the shore slightly later) than *E*. *radiata* (Stafford et al., 2012), which may reflect their higher thermal tolerance (Li, 2012), reducing the requirements for stress mitigation. The overall available window for mating is, therefore, severely restricted to rising tides that are high enough to reach the snails during the hot season (Ng et al., 2016). Stress mitigation activity (trail following for aggregation formation) as the tide falls, it would seem, overrides reproductive activity.

A significant proportion (*E*. *malaccana*, ~33% in summer and ~9% in winter; *E*. *radiata*, ~44% in summer and ~28% in winter) of the activity budget of both species was categorized as 'inactive' (stationary). While true in the sense of not moving, such 'inactivity' in snails can be regarded as a stress mitigation, especially during emersion. During emersion, these snails generally retract their foot and seal their shells with mucus, often attaching themselves to the rock with a mucus thread to reduce water loss and heat transfer from the rock surface (Miller & Denny, 2011; Vermeij, 1971), and may enter a phase of metabolic depression, which can function in energy saving (Marshall & McQuaid, 2011). This 'inactive' behavior, therefore, may in fact be associated with an active physiological mechanism to minimize energy expenditure.

In terms of the prevalence of stress‐mitigating behaviors, contrary to our preconceptions, the cool season appeared more 'stressful' for these snails than the hot season, as they spent most of the cool season sheltering in specific microhabitats (aggregations and crevices). We considered this 'sheltering' rather than a temperature‐based lack of function, since although these snails have reduced metabolism (20%–60% of maximum) in the cool season, their locomotory function is not greatly diminished (SLY Lau, unpublished data). Further, both food availability (Mak, 1996; Nagarkar & Williams, 1999) and the opportunity to feed (i.e. their potential window of activity, SLY Lau, unpublished data) are reduced in the cool season, due to the snails’ higher location on the shore. In this scenario, it is likely that energy maximization would be paramount, and consequently, moving and foraging would be expected, unless prevented by other factors. It is, however, likely that aggregation and crevice occupation behaviors perform different roles in the cool season as compared to the hot season. For example, evidence from more cool, temperate regions suggests that aggregations can actually raise the temperature of individuals in winter (Chapperon & Seuront, 2012). Aggregation and crevice occupation may also reduce the risk of dislodgement by wave action, which is on average significantly higher in the cool season on Hong Kong shores (Apps & Chen, 1973). As such, the function of these aggregations, allowing the snails to reduce activity, possibly depress their metabolism, and remain safe from high wave action, may be fundamentally different from their function in the hot season (see also Reid & Harley, 2021).

Previous physiological studies suggest that high‐shore species are likely to be early victims of climate change (Stillman, 2003; Stillman & Somero, 2000). Growing evidence (Marshall et al., 2013; Marshall, Mustafa, et al., 2010; Ng et al., 2017; Seuront & Ng, 2016), however, indicates that these high‐shore snails, as well as having high thermal tolerances, have developed a set of unique behavioral strategies to adapt to life in this extreme environment. Contrary to suggestions that evolution will have selected for thermal specialists to occupy extreme environments in the tropics (Tewksbury et al., 2008), with associated narrow thermal safety margins, the ability to invoke state‐dependent and often multifunctional behavioral strategies has the potential to widen thermal safety margins (see Ng et al., 2017) and strongly buffers environmental stress while achieving multiple benefits, allowing these snails to exploit this extreme niche. The lack of evolutionarily similar strategies in other groups may explain why tropical high‐shore environments, where the potential activity window is heavily constrained, are so species poor. The multifaceted behavioral repertoire of littorinids thus reveals the necessity of these overlooked, but novel adaptations as an evolutionary solution to persist in challenging and extreme environments.

## CONFLICT OF INTEREST

There are no conflicts of interest.

## AUTHORS CONTRIBUTION

**Terence Ng:** Conceptualization (equal); Formal analysis (equal); Investigation (equal); Methodology (equal); Writing‐review & editing (equal). **Sarah Lau:** Conceptualization (equal); Investigation (equal); Methodology (equal); Writing‐review & editing (equal). **Mark Davies:** Conceptualization (equal); Methodology (equal); Writing‐original draft (lead); Writing‐review & editing (lead). **Richard Stafford:** Conceptualization (equal); Methodology (equal); Writing‐review & editing (equal). **Laurent Seuront:** Conceptualization (equal); Methodology (equal); Writing‐review & editing (equal). **Neil Hutchinson:** Conceptualization (supporting); Methodology (supporting); Writing‐review & editing (equal). **Tin Yan Hui:** Formal analysis (equal); Writing‐review & editing (equal). **Gray Williams:** Conceptualization (equal); Methodology (equal); Writing‐original draft (lead); Writing‐review & editing (lead).

## Data Availability

The data are available at https://doi.org/10.5061/dryad.kh189325q.
